# Optical trapping reveals differences in dielectric and optical properties of copper nanoparticles compared to their oxides and ferrites

**DOI:** 10.1038/s41598-020-57650-2

**Published:** 2020-01-27

**Authors:** Pablo Purohit, Akbar Samadi, Poul Martin Bendix, J. Javier Laserna, Lene B. Oddershede

**Affiliations:** 1 Universidad de Málaga, Departamento de Química Analítica, Campus de Teatinos s/n, 29071 Málaga, Spain; 2 Niels Bohr Institute, University of Copenhagen, Blegdamsvej 17, 2100 Copenhagen, Denmark

**Keywords:** Nanoparticles, Optics and photonics

## Abstract

In a nanoplasmonic context, copper (Cu) is a potential and interesting surrogate to less accessible metals such as gold, silver or platinum. We demonstrate optical trapping of individual Cu nanoparticles with diameters between 25 and 70 nm and of two ionic Cu nanoparticle species, CuFe_2_O_4_ and CuZnFe_2_O_4_, with diameters of 90 nm using a near infrared laser and quantify their interaction with the electromagnetic field experimentally and theoretically. We find that, despite the similarity in size, the trapping stiffness and polarizability of the ferrites are significantly lower than those of Cu nanoparticles, thus inferring a different light-particle interaction. One challenge with using Cu nanoparticles in practice is that upon exposure to the normal atmosphere, Cu is spontaneously passivated by an oxide layer, thus altering its physicochemical properties. We theoretically investigate how the presence of an oxide layer influences the optical properties of Cu nanoparticles. Comparisons to experimental observations infer that oxidation of CuNPs is minimal during optical trapping. By finite element modelling we map out the expected temperature increase of the plasmonic Cu nanoparticles during optical trapping and retrieve temperature increases high enough to change the catalytic properties of the particles.

## Introduction

The unique plasmonic properties of metallic nanoparticles have placed them in a privileged spot among other materials in nanoscience^1–3^. Copper nanoparticles (CuNPs) have been proposed for a number of applications within photonics^4^, catalysis^5^, nanothermometry^6^, biosensing^7,8^ or nanomedicine^9^. Nevertheless, the choice of copper tends to fall behind silver, gold or platinum, probably due to its poor stability, i.e., its tendency towards oxidation. Spontaneous formation of Cupric oxide (CuO) and Cuprous oxide (Cu_2_O) leads to changes of plasmonic properties and chemical reactivity^10^ and is difficult to control. The Cu-containing ferrite CuFe_2_O_4_ (cuprospinel) and CuZnFe_2_O_4_ nanoparticles belong to the spinel group and derive from magnetite (Fe3O4) with Cu^+2^ and Zn^+2^ partially substituting Fe^+2^ in the crystalline structure. These particles are also of bio-medical interest since cuprospinel can be used as a cytotoxic agent in cancerous cells^11^ and CuZnFe_2_O_4_ has been proposed as an antibacterial agent^12^. Moreover, cuprospinel is a material of interest in photocatalytic production of hydrogen^13^.

In the aforementioned applications the interaction between the Cu nanoparticles and the incoming light is of crucial importance and in certain applications a controlled positioning of the particle is needed. Optical tweezers are a well-known tool by which nano- or micro-scopic particles can be manipulated and accurately positioned using a highly focused laser beam^14^. Not only dielectric particles but also plasmonic nanoparticles made of gold, silver or platinum have been reported individually optically trapped using a tightly focused Gaussian laser beam, one of the simplest implementations of optical tweezers^15–18^, and their interaction with electromagnetic field, including the absorption and scattering of the particles, is relatively well understood^19–23^. For copper, however, only larger particles, 1–20 µm, have been reportedly trapped^24^, and a quantitative description of the interaction between CuNPs and the electromagnetic field is still lacking.

The optical trap exerts a harmonic potential on the trapped particle and determination of the spring constant characterizing the potential provides a way to quantify how the particle interacts with the applied EM field. In the current manuscript we demonstrate optical trapping of Cu nanoparticles, their oxides and ferrites, and also use the tweezers to explore and quantify their interaction with the EM field. Our results demonstrate that even smaller changes of chemical bonding within metallic nanoparticles, or the presence of an oxide layer, have substantial effect on the particle’s optical properties. Moreover, using Finite Element Modelling (FEM) we calculated relevant optical cross-sections, the polarizability, as well as the expected heating of optically trapped CuNPs. The steep and highly spatially-controlable heating delivered by metallic nanoparticles is of significant interest for catalytic and chemical applications of CuNPs^4^.

## Results and Discussion

### Particle characterization and power spectra acquisition

**Figure 1:**
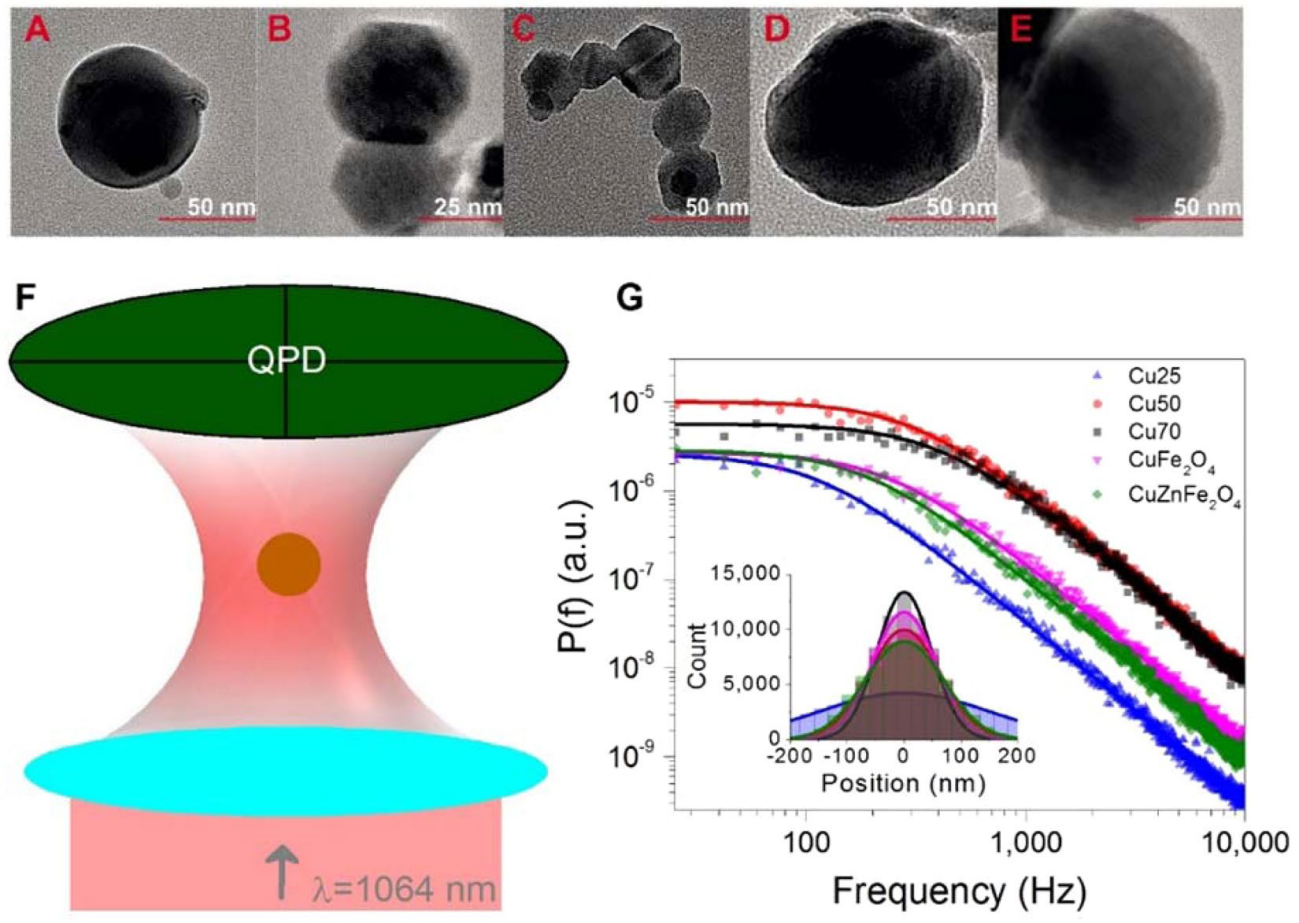
Electron microscopy images of (**A**) 70 nm, (**B**) 50 nm, (**C**) 25 nm, CuNP. (**D**) 90 nm CuFe_2_O_4_, and (**E**) 90 nm CuZnFe_2_O_4_, nanoparticles. (**F**) Schematic of the optical trapping experiment. (**G**) Power spectra, *P(f)*, of Cu25 (blue up-triangles), Cu50 (red circles), Cu70 (black squares), CuFe_2_O_4_ (magenta down-triangles) and CuZnFe_2_O_4_ (green diamonds) nanoparticles, trapped by 1064 nm laser light at P = 270 mW at the focal plane. Solid lines depict Lorentzian fits (Eq. 2) of the spectra using routine from ref. ^25^. Inset shows positional histograms for the trapped particles along with Gaussian fits (full lines).

Diluted suspensions of CuNPs with diameters of 25 nm (Cu25), 50 nm (Cu50) and 70 nm (Cu70), 90 nm (CuFe_2_O_4_), and 90 nm (CuZnFe_2_O_4_) were prepared as described in Methods. To evaluate particle morphology and size, TEM images (see equipment details in Methods) of these suspensions were acquired. Example images are shown in Fig. 1A–E and overall, particle sizes and morphologies were found to be in good agreement with those provided by the manufacturer. Figure 1F illustrates the experimental setup with which the particles were individually optically trapped by a tightly focused 1064 nm laser beam. The particle’s positional time series was acquired by a quadrant photodiode (QPD). The recordings on the QPD allowed for assessment of whether one or more particles were optically trapped^16^. Due to the high dilution, sonication and filtration of the sample, there was essentially no particle aggregation and most often only a single particle would be trapped; only time series originating from single particle trapping were analysed.

Optical tweezers exert a harmonic force on the trapped NP, ***F***_***trap***_ = *−κ****x***, where *κ* is the spring constant characterizing the optical trap and ***x*** is the deviation from the equilibrium position. Hence, if *κ* is known, the force acting on the particle can be found for any position. For this reason, the current manuscript aims at determining *κ* for optical trapping of CuNPs. The particle’s dynamics in any translational dimension is well described by the Langevin equation,

1 $$m{\boldsymbol{a}}\,{\boldsymbol{(}}{\boldsymbol{t}}{\boldsymbol{)}}=\,-\gamma {\boldsymbol{v}}\,{\boldsymbol{(}}{\boldsymbol{t}}{\boldsymbol{)}}-\,\kappa {\boldsymbol{x}}\,{\boldsymbol{(}}{\boldsymbol{t}}{\boldsymbol{)}}+\,{{\boldsymbol{F}}}_{thermal}{\boldsymbol{(}}{\boldsymbol{t}}{\boldsymbol{)}},$$

where *m* is the particle’s mass, ***a(t*)** its acceleration, ***v(t)*** its velocity, *γ* the friction coefficient, and ***F***_***thermal***_***(t)*** is a time-dependent stochastic force originating from thermal collisions within the medium. In water the particle’s motion is overdamped and the inertial term, *m****a(t)***, can be ignored. Hence, the positional power spectrum is given as

2 $$P\,(f)=\,\frac{{K}_{B}T}{\gamma }\,\frac{1}{{f}^{2}+\,{f}_{c}^{2}},$$

where *γ* = *6πŋR* for spherical particles (with *R* being the particle’s radius and *ŋ* the medium’s viscosity) and *f*_*c*_ = *κ/2πγ* denotes the corner frequency. Figure 1G shows power spectra of all trapped particles along with fits (full lines) of Eq. 2 using the routines described in ref. ^26^ which return values of *f*_*c*,_ and hence of *κ*, as further discussed in the following section. It is worth noticing that all obtained power spectra are well fitted by Eq. 2 and that the signal is entirely different from that of an empty trap (see Supporting Fig. S1), both in terms of signal amplitude and appearance.

The inset of Fig. 1G demonstrates that all position histograms are Gaussian, as expected because the particle is trapped in a harmonic potential. For pure CuNPs, the width of the histograms, and thereby the variance of the signal, increases with particle size as expected because the smaller the particle, the larger the excursions within the optical trap. For ferrites, the situation is more complex as not only their size but also their material properties change in comparison to the pure CuNPs.

### Optical trapping strength of individual CuNPs and ferrites depends on particle size and chemical nature

**Figure 2:**
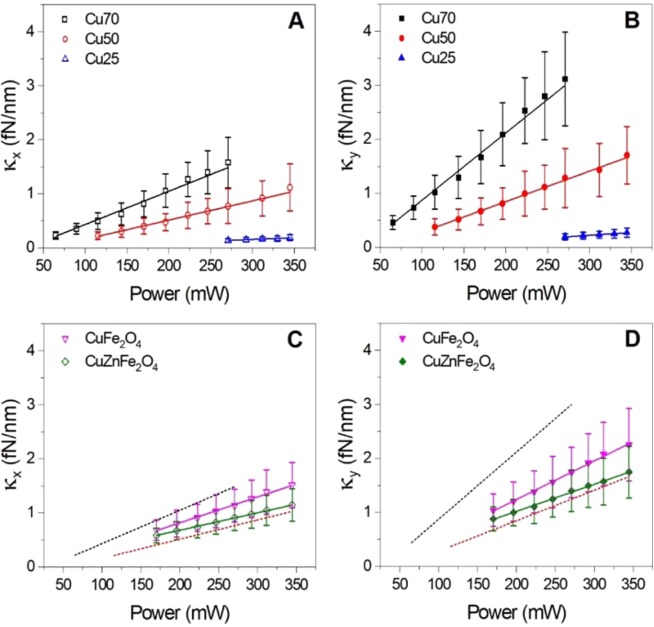
Trap stiffness for individual Cu, CuFe_2_O_4_ and CuZnFe_2_O_4_ nanoparticles versus laser power. (**A**, **B**) Stiffness in a direction parallel (**A**) or perpendicular (**B**) to the laser’s polarization for 70 nm (black squares), 50 nm (red circles), and 25 nm (blue triangles) CuNPs as a function of laser power at the sample plane. Lines show linear fits to data. (**C**,**D**) Same for 90 nm CuFe_2_O_4_ (magenta triangles) and 90 nm CuZnFe_2_O_4_ (green diamonds) nanoparticles. Lines show linear fits to data. In (**C**,**D**) the linear fits for 70 nm and 50 nm CuNPs from (**A**,**B**) are plotted with dashed black and red lines, respectively, to allow for easier comparison between the trapping stiffness of solid CuNPs and Cu ferrites.

The trap stiffnesses in the lateral plane *κ*_*x*_ (parallel to the laser’s polarization direction) and *κ*_*y*_ (orthogonal to the laser’s polarization direction) were extracted via power spectral analysis of the time series as described in the preceding section. Values of *κ* versus laser power are shown in Fig. 2A–D. For all particles, the trap stiffness increased linearly with laser power, a hallmark of successful optical trapping. As expected, the larger the solid CuNPs, the higher the spring constant, as observed also for solid gold and silver nanoparticles^16,17^.

The trap stiffnesses of CuFe_2_O_4_ and CuZnFe_2_O_4_ are shown in Fig. 2C,D. In this figure, the lines fitted to the experimentally obtained values of *κ* for Cu70 and Cu50 (from Fig. 2A,B) are shown as dashed black and red lines, respectively, to allow for easy comparison. At all laser powers, the trap stiffnesses for the 90 nm CuFe_2_O_4_ and CuZnFe_2_O_4_ nanoparticles turned out to be lower than those for the 70 nm CuNPs, despite their larger size. This can be attributed to the ionic chemical bonds which conform the oxides as they restrict the electron mobility through the crystalline structure in stark contrast to the delocalized electron clouds enclosing Cu-Cu bonds in the pure metallic particles, thus leading to a more limited particle-laser light interaction for the ferrites.

In order to compare *κ* for CuZnFe_2_O_4_ NPs versus *κ* for CuFe_2_O_4_ NPs at each laser power (as shown in Fig. 2C,D), we first used a D’Agostino-Pearson’s K-squared test to assess the normality of the measured distributions of *f*_*c*_ (which are directly proportional to *κ*) at each laser power. The numbers of individual particles measured at each laser power for CuFe_2_O_4_ and CuZnFe_2_O_4_ are given in Table S1. For each particle, 5 measurements were made, both of *f*_*cx*_ and of *f*_*cy*_. As all distributions were found to be normally distributed, we proceeded to perform a Two Sample t-test (including Welch’s correction for unequal variances) as well as a Two Sample Test for Variance (both with significance level = 0.05), in order to compare the two independent distributions of *f*_*c*_ for the two particle types measured at each laser power. For all laser powers, the average value of *f*_*c*_ is larger for CuFe_2_O_4_ particles than for CuZnFe_2_O_4_ particles. For laser powers of 223 mW and above, the two data sets representing CuFe_2_O_4_ and CuZnFe_2_O_4_ NPs, respectively, are significantly different (on a p = 0.05 significance level). For example, at a laser power of P = 223 mW Welch’s t-test returned a value of p = 0.030 when comparing *f*_*cy*_ values and a value of p = 0.031 when comparing *f*_*cx*_ values between the two particle types. As *κ* is directly proportional to *f*_*c*_ *(f*_*c*_ = *κ/2πγ)*, we find that at all laser powers average values of *κ* for CuFe_2_O_4_ particles are larger than for CuZnFe_2_O_4_ particles and for laser powers of 223 mW and higher, this difference is significant on a 0.05 significance level. This is true both in the *x* and *y* directions. A plausible reason for this measured difference in trapping strength could be that the Zn-O bond found in CuZnFe_2_O_4_ is more covalent than both the Cu-O and Fe-O bonds since Zn^+2^ features a less metallic behavior than the two other counter cations. This leads to an overall lower permittivity, thus explaining the lower trapping efficiency observed for the CuZnFe_2_O_4_ nanoparticles.

### Optical cross sections and polarizability of CuNPs

The size of the CuNPs here investigated is small enough that they belong to the Rayleigh regime. In the Rayleigh regime, the optical force can be written as ${\boldsymbol{F}}={{\boldsymbol{F}}}_{grad}+{{\boldsymbol{F}}}_{scat}$^26,27^, where the gradient force, responsible for optical trapping, is given as:

3 $${{\boldsymbol{F}}}_{grad}=\,\frac{{\alpha }_{r}}{4}{\rm{\nabla }}\langle {|{\boldsymbol{E}}|}^{2}\rangle \cdot $$

Here, *α*_*r*_ is the real part of the polarizability and ***E*** is the electric field of the trapping laser.

The scattering force, ***F***_***scat***_, destabilizing the optical trap, includes both radiation pressure and spin curl force and is given as:

4 $${{\boldsymbol{F}}}_{{\boldsymbol{s}}{\boldsymbol{c}}{\boldsymbol{a}}{\boldsymbol{t}}}={C}_{ext}\left\{\frac{1}{c}\langle {\boldsymbol{S}}\rangle +c{\rm{\nabla }}\,\times \,\langle {{\boldsymbol{L}}}_{{\boldsymbol{s}}}\rangle \right\}$$

where *C*_*ext*_ is the extinction cross-section of the particle, 〈**S**〉 is the time averaged Poynting vector, *c* is the speed of light, and 〈***L***_*s*_〉 is the time averaged spin density of the electromagnetic field, $\langle {{\boldsymbol{L}}}_{s}\rangle =\frac{{\varepsilon }_{0}}{4\omega i}\{{\boldsymbol{E}}\,\times \,{{\boldsymbol{E}}}^{{\boldsymbol{\ast }}}\}$. The extinction cross-section is a sum of the scattering and absorption cross-sections: ${C}_{ext}=\,{C}_{scat}+{C}_{abs}$.

The particle’s complex polarizability, *α* = *α*_*r*_ + *iα*_*i*_, is related to its absorption and scattering cross-sections:

5 $${C}_{abs}=\frac{k}{{\varepsilon }_{0}}{\alpha }_{i}$$

6 $${C}_{scat}=\frac{{k}^{4}}{6\pi {\varepsilon }_{0}^{2}}\,{|\alpha |}^{2}$$

where *k* = *2πn*_*m*_*/λ*_0_, is the wavenumber, *n*_*m*_ is the refractive index of the medium, and *λ*_0_ the wavelength in vacuum.

As can be seen from Eq. 3, the gradient force is proportional to the real part of the particle’s polarizability, *α*_*r*_, which can be found from Eqs. 5 and 6 if the cross sections are known. Equation 4 shows that the destabilizing scattering force is proportional to *C*_*scat*_ + *C*_*abs*_. In order to obtain stable 3D trapping, the gradient forces must balance or overcome the scattering force. The absorption cross section, *C*_*abs*_, provides information on the amount of energy absorbed by the particle and is directly related to the associated temperature increase.

To calculate *C*_*scat*_ and *C*_*abs*_ for the massive CuNPs, we used finite element modeling (FEM) implemented in COMSOL software to solve the scattering problem in the frequency domain by numerically solving Maxwell’s equations within a discretized space. This procedure has previously been employed to calculate optical properties of metallic nanoparticles and has been shown to provide results reproducing direct experimental measurements^20,21^. We used tetrahedral meshes with a mesh size of 1/10 of the particle diameter. This is much below the threshold where further minimizing the mesh size does not change the results. By FEM we found the electric field, ***E***, satisfying the boundary condition

7 $$\nabla \times {\mu }_{r}^{-1}(\nabla \times {\boldsymbol{E}})-{k}_{0}^{2}\left({\varepsilon }_{r}-i\frac{\sigma }{\omega {\varepsilon }_{0}}\right){\boldsymbol{E}}=0.$$

Here, ${\boldsymbol{E}}={{\boldsymbol{E}}}_{inc}+{{\boldsymbol{E}}}_{scat}$, is a sum of the incoming and scattered electric fields. ${\mu }_{r}$ and ${\varepsilon }_{r}$ are the frequency dependent relative permeability and permittivities, respectively, *ω* is the angular frequency, $\sigma$ is conductivity and ${k}_{0}^{2}=\frac{{\omega }^{2}}{{c}^{2}}$. All optical constants and wavelength dependent Cu permittivities were deduced from fitting the Brendel-Bormann model to data^22,23^. For CuO, the wavelength dependent permittivities were provided by Nanocomposix, Czech Republic.

From knowledge of ***E*** the absorption and scattering cross-sections can be calculated^15^

8 $${C}_{abs}=\frac{2}{c{\varepsilon }_{0}{|{{\boldsymbol{E}}}_{inc}|}^{2}}\iiint q\,dV=\,\frac{2}{c{\varepsilon }_{0}{|{{\boldsymbol{E}}}_{inc}|}^{2}}\iiint \frac{1}{2}Re({\boldsymbol{J}}\,\cdot {{\boldsymbol{E}}}^{\ast })\,dV$$

9 $${C}_{scat}=\frac{2}{c{\varepsilon }_{0}{|{{\boldsymbol{E}}}_{inc}|}^{2}}\iint ({\boldsymbol{n}}\cdot {{\boldsymbol{S}}}_{scat})dS=\frac{2}{c{\varepsilon }_{0}{|{{\boldsymbol{E}}}_{inc}|}^{2}}\iint \frac{1}{2}[n\cdot Re({{\boldsymbol{E}}}_{scat}\,\times \,{{\boldsymbol{H}}}_{scat}^{\ast })]\,dS$$

where ***S***_*scat*_ = $\frac{1}{2}Re({{\boldsymbol{E}}}_{scat}\,\times \,{{\boldsymbol{H}}}_{scat}^{\ast })$ is the scattered Poynting vector along the normal vector, ***n***, in the outwards direction from the scatterer’s surface that delimits the integral. The power loss density, *q*, is given by $q=\frac{1}{2}Re({\boldsymbol{J}}\cdot {{\boldsymbol{E}}}^{\ast })$, where ${\boldsymbol{J}}={\rm{\sigma }}\,{\boldsymbol{E}}$ is the current density within the nanoparticle. Complex conjugates are denoted with an asterisk.

**Figure 3:**
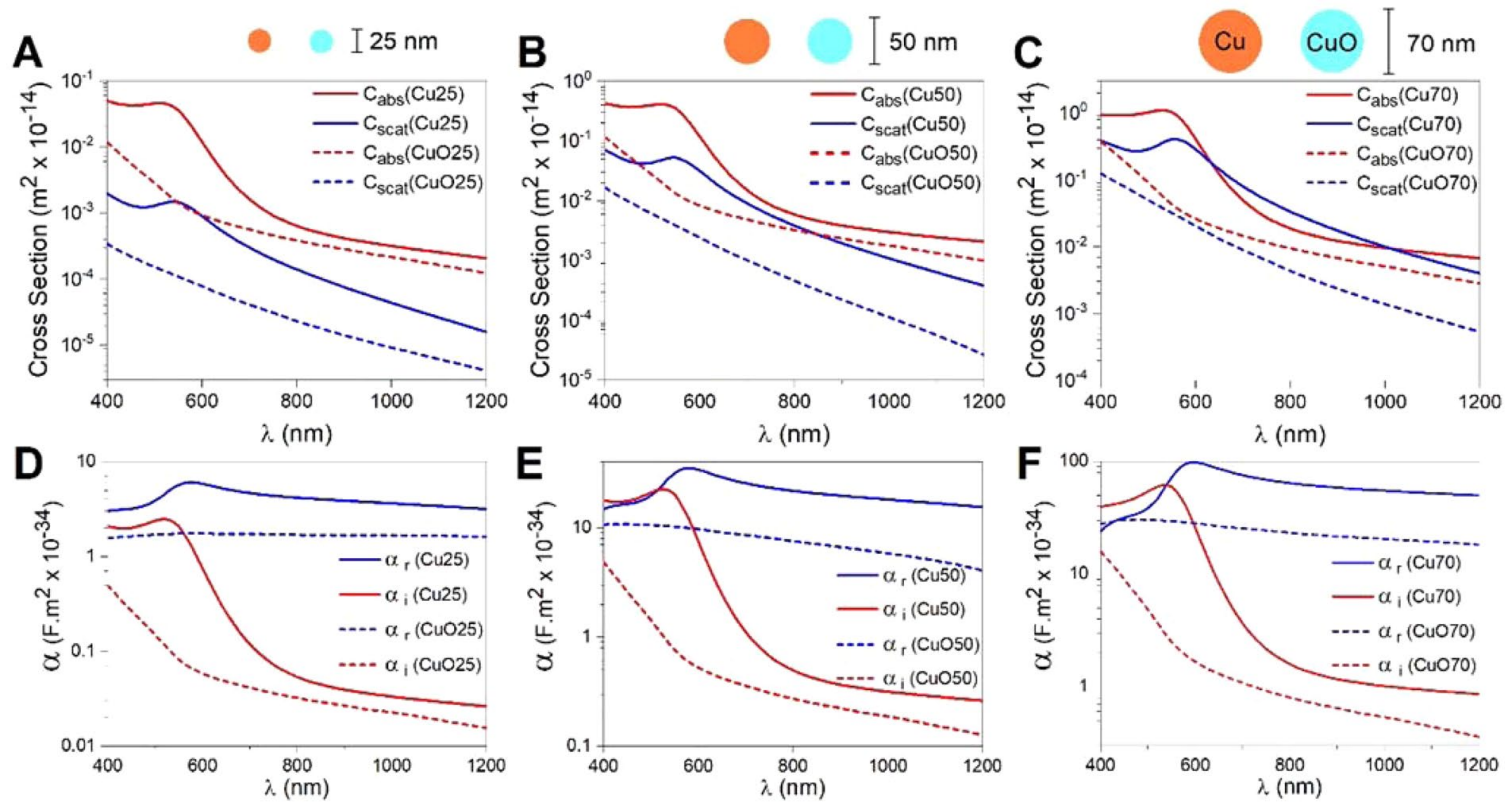
Absorption (red) and scattering (blue) cross-sections as a function of wavelength calculated by FEM for (**A**) 25 nm, (**B**) 50 nm, (**C**) 70 nm CuNPs (solid lines) and CuONPs (dashed lines). Real (blue) and imaginary (red) parts of polarizability versus wavelength for, (**D**) 25 nm, (**E**) 50 nm, (**F**) 70 nm CuNPs (solid lines) and CuONPs (dashed lines). Semi-log scales were employed to magnify the behaviour in the NIR region.

The calculated optical cross sections, *C*_*abs*_ and *C*_*scat*_, are shown in Fig. 3A–C as a function of wavelength both for massive CuNPs and for fully oxidized CuNPs, CuONPs, of different sizes. For all wavelengths, both absorption and scattering cross sections are significantly higher for pure CuNPs than for CuONPs. Exact values for *C*_*scat*_ and *C*_*abs*_ at the optical trapping wavelength, 1064 nm, are given in Supporting Table S2.

The real and imaginary parts of the polarizability of CuNPs and CuONPs are shown in Fig. 3D–F. From Eq. (3) it is clear that *α*_*r*_ > 0 is a necessary condition for stable optical trapping. Hence, both CuNPs and CuONPs should be trappable within the whole wavelength window theoretically investigated, however, with CuNPs trapping more strongly than CuONPs, as experimentally observed for λ = 1064 nm (Fig. 2). Values for *α*_*r*_ and *α*_*i*_ at λ = 1064 nm are provided in Supporting Table S2.

As the gradient force, responsible for optical trapping, is directly proportional to *α*_*r*_ (see Eq. 3), we used the ratios of *α*_*r*_ and of *κ* for different particle types to compare the calculated expectations for trapping strength to the measured values. This is not a perfect comparison as the scattering force will also contribute to the total force. However, in this regime the gradient force dominates the scattering force, therefore, we calculated the polarizability ratios α_r,Cu1_/α_r,Cu2_ and compared these to the corresponding ratios of the measured trap stiffnesses, κ_Cu1_/κ_Cu2_. These ratios, given in Table 1, demonstrate a good agreement between predicted increase of trapping stiffness as a function of particle size for massive CuNPs and the experimental findings.

**Table 1:** Comparison of experimentally obtained trap stiffness ratios and the corresponding theoretically calculated ratios of the real part of the particles’ polarizability, *α*_*r*_, for different solid CuNPs.

*κ*_y_ ratio	*κ*_y_ ratio values	*α*_*r*_ ratio	*α*_*r*_ ratio values
${\kappa }_{y(Cu70)}/{\kappa }_{y(Cu50)}$	**2**.**5**	${\alpha }_{Cu70}/{\alpha }_{Cu50}$	**3**
${\kappa }_{y(Cu70)}/{\kappa }_{y(Cu25)}$	**16**	${\alpha }_{Cu70}/{\alpha }_{Cu25}$	**15**
${\kappa }_{y(Cu50)}/{\kappa }_{y(Cu25)}$	**6**.**5**	${\alpha }_{Cu50}/{\alpha }_{Cu25}$	**5**

The gradient force, responsible for optical trapping, is directly proportional to *α*_*r*_, therefore, the ratios of *κ* and *α*_*r*_ for different particle sizes are expected to be similar.

### Effect of oxidized shell on optical properties of CuNPs

**Figure 4:**
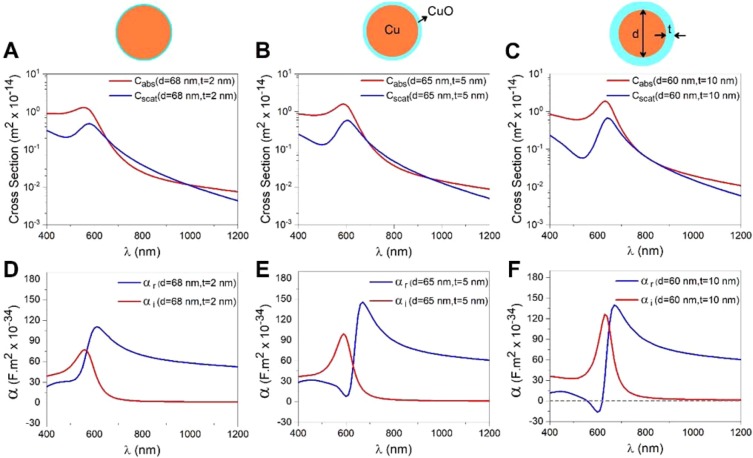
Absorption cross sections and polarization for CuNPs with an oxidized shell of varying thickness calculated by FEM. (**A–C**) Absorption (red), scattering (blue) cross-sections for CuNPs with an oxidized shell as a function of wavelength. The semi-log scales were employed to magnify the behaviour in the NIR region. (**D–F**) Real (blue) and imaginary (red) parts of polarizability versus wavelength. (**A**,**D**) Cu core: d = 68 nm, oxidized shell: t = 2 nm, (**B**,**E**) Cu core; d = 65 nm, oxidized shell: t = 5 nm, (**C**,**F**) Cu core: d = 60 nm, oxidized shell: t = 10 nm.

To further explore the effects of oxidization, we performed FEM calculations of CuNPs with an oxidized shell of varying thickness. When Cu oxidizes, it swells, hence, in our simulations, we assumed that half the CuO shell thickness is taken from the CuNP due to the oxidation and the other half is caused by swelling of the oxide layer. For example, in Fig. 4A, we consider a CuNP which originally had d = 70 nm; 1 nm of this particle oxidizes, which causes an additional swelling of 1 nm, hence, the total thickness of the oxide shell is 2 nm. In consequence, the Cu core has d = 68 nm and the overall diameter of the particle including the oxidized shell is 72 nm. FEM results for three different core-shell sizes of Cu-Cu_x_O NPs are presented in Fig. 4. It is clear that core-shell NPs with thin CuO shells have optical cross-sections and polarizabilities of similar magnitudes as CuNPs of same size (Fig. 3). Moreover, thicker CuO layers lead to a significant change of optical properties.

Experimentally we have seen no evidence in the TEM images (as, e.g., shown in Fig. 1A–E) of a swelled oxide shell. Hence, if such an oxide shell was present, it was either rather thin or invisible in the TEM images.

As the gradient force, responsible for optical trapping, is directly proportional to *α*_*r*_ (cf. Eq. 3), trapping will not be possible at wavelengths where *α*_*r*_ is very small or negative. Figure 4F illustrates such a scenario: for a shell thickness of 10 nm, α_r_ is negative around λ = 600 nm and such particles are hence not likely to be trappable at 600 nm. The real part of the polarizability, *α*_*r*_, for pure CuNPs, is more than twice that of a fully oxidized NP (Table S2) and, hence, the effect of oxidation could significantly affect the trapping efficiency of the NPs. As the ratios of the calculated polarizabilities for massive and pure CuNPs compared well to the ratios of the experimentally determined trap stiffnesses (Table 1), it is consistent with our experimental measurements if the CuNPs acquired only a shallow oxide layer.

The altered optical trapping properties of CuNPs as a function of their oxidation imply that the degree of oxidation of CuNPs could, in principle, be explored by trapping the CuNPs at specific wavelengths. For instance, one could choose laser lines in the interval 600–700 nm where *α*_*r*_ changes significantly with the thickness of the oxide layer.

### Plasmonic heating of irradiated CuNPs

Absorption of laser light by the trapped CuNPs will lead to heating of the particles with the temperature increase of a particle being proportional to *C*_*abs*_ and laser intensity^28^. This will naturally lead to an increase in the temperature of the media surrounding the particle which affects the viscosity of the medium and hence the motion of the particle. This phenomenon is known as Hot Brownian Motion^29,30^ and has implications for the dynamics of the particle and, hence, for its optical trapping properties.

**Figure 5:**
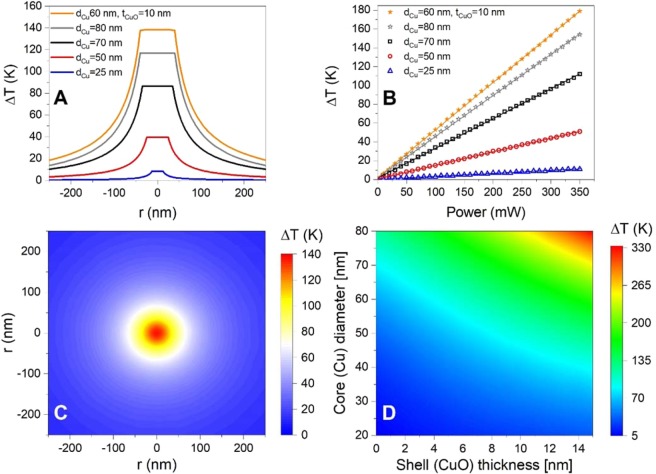
Theoretical calculation of plasmonic heating of trapped solid CuNPs and CuNPs with an oxidized shell. (**A**) Temperature profile around 25 nm (blue), 50 nm (red), 70 nm (black), and 80 nm (grey) solid CuNPs, as well as for a CuNP with an oxidized shell (core: d = 60 nm, shell: t = 10 nm) under the same conditions as in the optical trapping experiments. (**B**) Temperature increase as a function of laser power for the same particles as shown in (**A**). (**C**) 2D heat profile around a CuNP with an oxidized shell (core: d_Cu_ = 60 nm, shell: t = 10 nm). (**D**) Temperature increase for CuNPs with an oxidized shell for varying sizes of the diameter of the core and the thickness of the oxidized shell. For these calculations, λ = 1064 nm and P = 270 mW.

To estimate the temperature increase of an optically trapped CuNP we used FEM to theoretically predict its plasmonic heating in the laser trap. The temperature of an optically trapped plasmonic nanoparticle equilibrates within nanoseconds^21^, hence, at the timescales here considered, the temperature can be assumed constant across the particle and decays with distance to the particle’s surface, *r*, as^28,31^

10 $$\Delta T(r)=\,\frac{{C}_{abs}I}{4\pi Kr},r > R$$

where *I* is the trapping laser intensity, *R* is the radius of nanoparticle, and *K* is the thermal conductivity of surrounding medium (water). To find *C*_*abs*_ for each particle type we used FEM as described in a preceding section. Figure 5A shows the temperature profiles for three different sizes of optically trapped CuNPs calculated via Eq. (5); for these calculations a constant laser power of 270 mW is used as this is the highest laser power used in the optical trapping experiments for all particle types (as shown in Fig. 2). Also, the temperature profile for a CuNP with an oxidized shell (core: d_Cu_ = 60 nm), shell: t_CuO_ = 10 nm) is presented (orange line in Fig. 5A,B). The larger the particle, the higher its temperature. Equation 10 predicts a linear relation between temperature increment and laser power, this is shown in Fig. 5B for the same NPs featured in Fig. 5A. Interestingly, the particle with an oxidized shell (orange lines in Fig. 5A,B, core = 60 nm, shell: t = 10 nm) has a temperature that is higher than a similarly sized massive CuNP (80 nm, grey).

Figure 5C shows the 2D temperature profile around an irradiated CuNP with an oxidized shell (core: d_Cu_ = 60 nm, shell:t = 10 nm). Interestingly, varying the volume ratio between the Cu core and the oxidized shell can give rise to several hundreds of degrees of difference in the surface temperature at constant laser power.

In the calculations the particle is assumed to be located in the focus of a laser beam with a perfect Gaussian intensity profile. However, this is not a realistic experimental situation; inherent spherical aberration will inevitably cause distortions of the intensity profile, distortions which are significant at the nanometer scale. Also, optically trapped metallic nanoparticles have been shown to stably trap at positions far from the focus, either below or above, depending in a non-trivial manner on the particle size, material and laser power^32^. These effects will cause the laser intensity at the position of the particle to be unpredictable and, probably, substantially different than in the theoretical situation.

Chemical reaction rates are sensitively dependent on temperature as quantified by the Arrhenius equation. Therefore, heating of CuNPs is likely to be of crucial importance for their catalytic activity^4^. In accordance with this, previous studies have demonstrated that the oxidation state of Cu can be modified by laser light^33,34^.

## Conclusions

We demonstrated stable optical trapping of individual Cu, CuFe_2_O_4,_ and CuZnFe_2_O_4_ nanoparticles with diameters in the range of 25–90 nm using NIR light. We found that Cu-ferrites had a weaker interaction with the optical trap compared to CuNPs of similar sizes, which can be attributed to the ionic character of the oxides compared to the metallic character of pure Cu particles. By finite element modelling we calculated the optical properties of CuNPs and their oxides and showed that the presence of an oxidized shell can significantly change the optical properties of CuNPs. By tuning the ratio of the Cu core and the thickness of the oxidized shell one can obtain a situation where the real part of the polarizability becomes negative at certain wavelengths, thus rendering the particles non-trappable. Our experimental optical trapping measurements of solid CuNPs in water, however, indicated only minor to no oxidation of the surface. Due to the plasmonic nature of CuNPs, they are expected to heat significantly in the optical trap. Our theoretical calculations show that, if trapped in the focus of the laser beam, temperature increases up to hundreds of degrees Celsius are to be expected, with the exact value being crucially dependent on the particle’s size, oxidation state and position. This temperature increase is likely to affect CuNPs’ catalytic properties. Also, heating might cause ablation of the oxide layer and restore the physicochemical properties of the pure CuNP. Hence, laser induced heating may allow for remote control over the particle’s chemical and catalytic properties.

## Methods

### Optics and data acquisition

An inverted microscope (LEICA DMIRB HC) was used to integrate the optical trap, based on a CW Nd:YVO_4_ laser (λ = 1064 nm, 5 W, Spectra Physics). The laser was focused by an oil immersion objective (HCX, PL, APO, 100×/N.A. = 1.4). The immersion oil had an index of refraction of *n* = 1.54, ensuring minimum spherical aberration at the sample plane, located approximately at 5 µm from the chamber’s lower surface. A quadrant photodiode (QPD) (S5981, Hamamatsu) was placed in the conjugate plane of the condenser’s back focal plane to monitor the trapped particle position. To visualize the trapped particles, a CCD camera (25 Hz frame rate, Sony XC-EI50) was used to detect the backscattered light. When a particle was trapped, we monitored a significant change in the QPD signal; the amplitude of the time series, signifying the variance of the positions visited, increased to a steady value upon successful trapping. Should a second particle enter the trap, the signal on the QPD would again change as described in ref. ^16^. Also, we performed real-time calculations of the power spectrum of the QPD signal and the characteristic Lorentzian spectrum as displayed in Fig. 1G would only emerge when a particle was successfully trapped. A custom-made Labview program was used for processing the time series acquired by the QPD.

### Sample preparation

Dry nanopowdered Cu NPs with mean diameters of 25 nm (Cu25), 50 nm (Cu50) and 70 nm (Cu70) as well as 90 nm in diameter CuFe_2_O_4_ and CuZnFe_2_O_4_ NPs were purchased from mkNano (Canada). A small portion of the powders (below 1 mg) was suspended in 1.5 ml of ultrapure milliQ water and sonicated for ~20 minutes and then filtered through 100 nm pore diameter membranes to reduce the possible presence of clusters in the trapping chambers. Perfusion chambers were built by using a microscope slide and a cover glass separated by double-sided scotch tape. The volume of the chamber was completely filled with the diluted nano-suspensions.

### TEM imaging

The TEM images of the different particle types shown in Fig. 1A–E were obtained using a transmission electron microscope (Philips CM-12 (120 kV) and Philips EM-420 (120 kV) located at the University of Copenhagen.

## Supplementary information

Supporting Information.
