# Supplementary material for: Optical trapping reveals differences in dielectric and optical properties of copper nanoparticles compared to their oxides and ferrites
Source: Sci Rep. 2020 Jan 27;10:1198. doi: 10.1038/s41598-020-57650-2 (PMC6985125; doi:10.1038/s41598-020-57650-2)
Supplement: Supplementary file 1 — Supporting Information. [file 41598_2020_57650_MOESM1_ESM.pdf]

Supporting information for

"Optical trapping reveals differences in dielectric and optical properties of copper nanoparticles compared to their oxides and ferrites''

Pablo Purohit,[†a] Akbar Samadi,[†b] Poul Martin Bendix,[b] J. Javier Laserna,[a] and Lene B. Oddershede[*b]

[a] Universidad de Málaga, Departamento de Química Analítica, Campus de Teatinos s/n, 29071 Málaga, Spain

[b] Niels Bohr Institute, University of Copenhagen, Blegdamsvej 17, 2100 Copenhagen, Denmark

† These authors contributed equally and are joint first authors

*Correspondence: oddershede@nbi.ku.dk

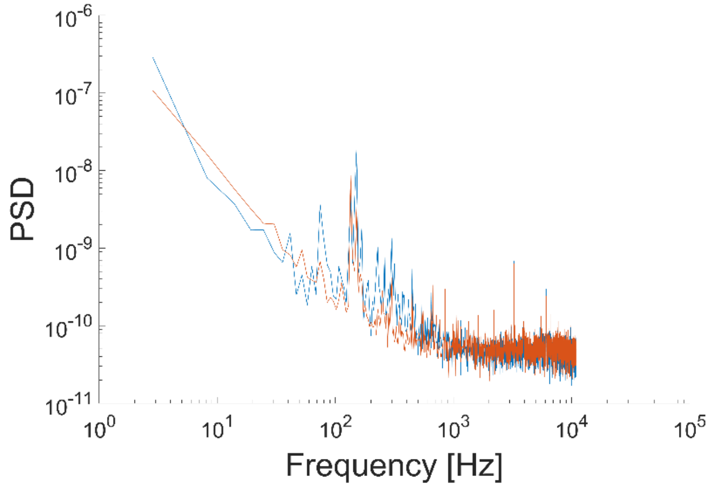

Figure S1. Power spectrum of signal in the lateral directions from the quadrant photodiode (QPD) in the case of an empty trap. Laser power: 240 mW. The amplitude of the signal is several orders of magnitude smaller than when a particle is trapped (see Figure 1G) and the signal from an empty trap does not have the characteristic Lorentzian shape expected for successful particle trapping (see Figure 1G).

Laser Power (mW)	n, $CuFe_2O_4$	n, $CuZnFe_2O_4$
**170**	17	8
**196**	17	9
**223**	17	13
**247**	17	13
**271**	17	13
**293**	16	13
**312**	16	13
**345**	13	13

Table S1: Number of individual particles, n, trapped and measured at each laser power for $CuFe_2O_4$ and $CuZnFe_2O_4$. For each particle, five measurements were made, both of f_{cx} and f_{cy}. All laser powers stated are at the sample plane, in accordance with Figure 2 of the main manuscript.

Sample	C_{abs} (m^2)	C_{scat} (m^2)	α_r (F . m^2)	α_i (F . m^2)
Cu70	8.50 x 10^{-17}	7.44 x 10^{-17}	5.33 x 10^{-33}	9.5 x 10^{-35}
Cu50	2.64 x 10^{-17}	7.83 x 10^{-18}	1.73 x 10^{-33}	2.97 x 10^{-35}
Cu25	2.75 x 10^{-18}	3.20 x 10^{-19}	3.50 x 10^{-34}	3 x 10^{-36}
CuO70	4.26 x 10^{-17}	1 x 10^{-17}	1.99 x 10^{-33}	4.78 x 10^{-35}
CuO50	1.5 x 10^{-17}	7.5 x 10^{-19}	5.36 x 10^{-34}	1.68 x 10^{-35}
CuO25	1.82 x 10^{-18}	7.23 x 10^{-20}	1.66 x 10^{-34}	2 x 10^{-36}
CuNP with oxidized shell (core: d=68nm, shell: t=2nm)	9.92 x 10^{-17}	8.16 x 10^{-17}	5.58 x 10^{-33}	1.11 x 10^{-34}

Table S2: FEM calculated values of optical properties at the trapping wavelength (λ =1064 nm).
